# Foliar Pre-Treatment with Abscisic Acid Enhances Olive Tree Drought Adaptability

**DOI:** 10.3390/plants9030341

**Published:** 2020-03-08

**Authors:** Cátia Brito, Lia-Tânia Dinis, Helena Ferreira, José Moutinho-Pereira, Carlos M. Correia

**Affiliations:** CITAB-Centre for the Research and Technology of Agro-Environmental and Biological Sciences, Universidade de Trás-os-Montes e Alto Douro, 5000-801 Vila Real, Portugal; cvqbrito@utad.pt (C.B.); liatdinis@utad.pt (L.-T.D.); helenaf@utad.pt (H.F.); moutinho@utad.pt (J.M.-P.)

**Keywords:** antioxidants, growth, minerals, mitigation strategies, oxidative stress, photosynthesis, recovery, water deficit

## Abstract

Water is the most widely limiting factor for plants distribution, survival and agricultural productivity, their responses to drought and recovery being critical for their success and productivity. *Olea europaea* L. is a well-adapted species to cyclic drought events, still at considerable expense of carbon reserves and CO_2_ supply. To study the role of abscisic acid (ABA) as a promoter of drought adaptability, young potted olive trees subjected to three drought-recovery cycles were pre-treated with ABA. The results demonstrated that ABA pre-treatment allowed the delay of the drought effects on stomatal conductance (g_s_) and net photosynthesis (A_n_), and under severe drought, permitted the reduction of the non-stomatal limitations to A_n_ and the relative water content decline, the accumulation of compatible solutes and avoid the decline of photosynthetic pigments, soluble proteins and total thiols concentrations and the accumulation of ROS. Upon rewatering, ABA-sprayed plants showed an early recovery of A_n_. The plant ionome was also changed by the addition of ABA, with special influence on root K, N and B concentrations. The improved physiological and biochemical functions of the ABA-treated plants attenuated the drought-induced decline in biomass accumulation and potentiated root growth and whole-plant water use efficiency after successive drought-rewatering cycles. These changes are likely to be of real adaptive significance, with important implications for olive tree growth and productivity.

## 1. Introduction

On global basis, water is the most widely limiting factor for plants distribution, survival and agricultural productivity. Water deficit occurs when there is not enough water to absorb in order to replace the losses by transpiration, or when plants encounter environmental conditions that hinder the absorption process [1]. Consequently, plants present a lower amount of water relatively to a state of maximum hydration, impairing a variety of physiological and biochemical responses at the cellular and organism levels [1]. Thus, the responses and adaptation of species to drought are critical for their success in any environmental niche and for their use and productivity in agricultural ecosystems [2]. In addition, the capacity to recover the plant functions when water shortage is relieved is crucial to restart growth under cyclic drought events. Recovery after stress is a very complex process involving the rearrangement of many metabolic pathways to repair drought-induced damages and resume plant growth and gain yield [3]. That is why Chen et al. [3] draw attention to the concept of “drought adaptability”, which integrates drought resistance and recovery capacity.

Olive tree (*Olea europaea* L.) is a woody species which grows under the typical Mediterranean semi-arid conditions, a region already affected by multiple environmental constraints factors, and particularly susceptible to climate change, being expected higher temperatures and shifts in the precipitation patterns, leading to higher evaporative demand and lower soil water availability [4]. Although olive is a crop well-adapted to harsh conditions, water deficit has negative repercussions on water relations, carbon assimilation, oxidative pathways, nutrient uptake and biomass accumulation [1,5,6,7,8,9,10]. Moreover, the adaptation mechanisms adopted by this species against drought stress are activated at the expense of carbon reserves and may be detrimental with the increased duration and intensity of stress. The increasing consciousness regarding the nutritional value of olive oil has enhanced the demand for this product and, thus, it is crucial to increase olive trees ability to conserve and use the scarce available water and the low and unexpected rainfall during the summer season.

Abscisic acid (ABA) is a well-known plant stress hormone, being assumed as a potential mediator for induction of drought tolerance in plants [2]. Under drought, ABA elicits two distinct responses, where the earliest and most rapid is stomatal closure, which minimizes the water loss through transpiration. Further, ABA gradually increases hydraulic conductivity and promotes root cell elongation, enabling the plant to recover, and inducing the accumulation of osmotically active compounds, which protects cells from damage [11]. In addition, the regulation of antioxidant responses is linked to ABA signalling pathways [12,13,14]. Although ABA-induced stomatal closure allowed water conservation, this comes at the expense of CO_2_ supply to photosynthesis [15]. Still, the opposite can also be true, when the positive influence of ABA in alleviation of non-stomatal limitations surpass the effect of stomatal closure [13,16]. For that reason, the exogenous application of ABA has been associated with drought tolerance promotion [12,13,14,15,16,17,18,19,20]. However, it is not easy to deduce a physiological role for any naturally occurring endogenous compound from its exogenous application [21], as it might depends on stress level, species, genotypes, vegetative stage, and dose, time, method and frequency of application [12,13,14,15,16,20,22,23,24].

Foliar spray was the chosen method to be evaluated in this study, as can generate a faster plant response in terms of saving water and stimulating the antioxidant system [13]. As far as we are aware, no studies evaluated the effects of foliar pre-treatment with ABA on plant responses during subsequent drought events and on the capacity for recovery after rehydration in outdoor conditions. As an outdoor pot experiment, plants can grow under near-natural conditions, thus, the results of this experiment are better transferable to field grown plants [25]. Due to the huge economic and ecological importance of olive tree and the role of ABA in regulating plant water relations, we aim to evaluate how a foliar application of ABA impacts the drought and recovery responses of olive plants. For this propose, we analysed the effects of ABA application on (i) physiological and biochemical variables under drought and recovery upon rewatering; (ii) on mineral status (iii) and on growth responses.

## 2. Materials and Methods

### 2.1. Plant Material and Experimental Set-Up

The experiment was carried out in Vila Real, Northeast Portugal, with own-rooted 3-years-old olive trees (*Olea europaea* cv. Cobrançosa). Details of growth conditions can be found in [8]. Prior to the experiment, fifty-six uniform plants, selected based on height, leaf number and leaf area were left for 30 days in the study site for acclimatization. Then, at the beginning of the experiment, eight plants randomly chosen were harvested to assess the initial biomass of the different plant organs. The remaining forty-eight plants were divided in three groups, each one comprising sixteen plants. One group was sprayed with distilled water and kept under well-watered conditions (WW) thought the entire experimental period, in which plants were watered every day, till field capacity. The other two groups were subjected to three “drought-rewatering cycles” by withholding water until the occurrence of precipitation (1st and 2nd cycles), or until the stomatal conductance for water vapour during midmorning (peak of photosynthetic activity) dropped around 50 mmolm^−2^ s^−1^ (reached at 3rd cycle), as reported elsewhere [8]. From this two groups, one was sprayed with distilled water (D) and the other with 80 µM abscisic acid (ABA) (D + ABA). All spray applications were supplemented with 0.1% (*v/v*) Tween 20 and conducted according to good efficacy practice standard operating procedures adjusted for agricultural experiments. Care was taken during the application of foliar sprays to avoid overspraying non-target trees, covering them with a plastic sheet. The 1st, 2nd and 3rd “drought-re-watering cycles” had the duration of 12–6 days, 9–3 days and 21–16 days, respectively.

**Figure 1:**
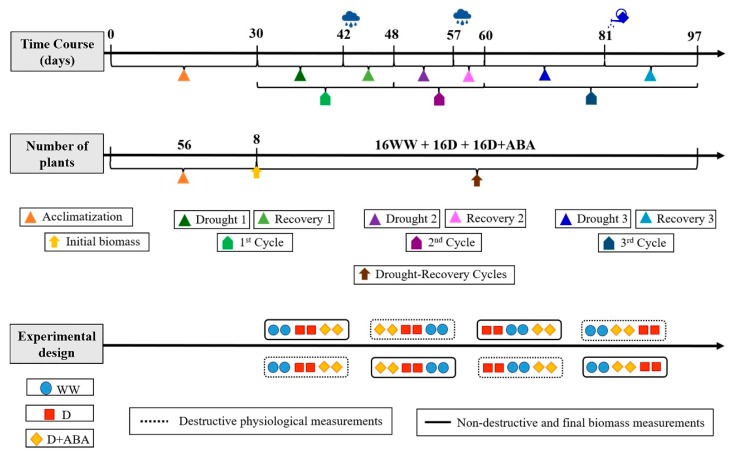
Schematization of the experiment. Well-watered (WW) and droughted without (D) and with abscisic acid (D + ABA) plants.

Each group of sixteen plants was divided in two subgroups, each one with eight plants. Plants from one subgroup were used for physiological and biochemical measurements, while plants from the other subgroup were used for final biomass assessment and mineral analysis. A schematic representation of the experiment is presented in Figure 1.

All physiological and biochemical measurements at leaf level were measured in healthy, full expanded mature leaves. The daytime leaf gas exchange and leaf relative water content measurements (*n* = 8) were taken periodically during the three drought-recovery cycles. Leaf samples for biochemical analysis (*n* = 8) were taken at the peak of severest drought period (DP) (3rd cycle) and eight days after the respective recovery period (RP). For growth, biomass accumulation, plant organs ionome and whole-plant water use efficiency (*n* = 8), plants were harvested at the end of the experiment.

### 2.2. Leaf Water Status

Leaves detached were immediately placed into air-tight containers and then the following parameters were examined: fresh weight (FW, g); weight at full turgor (TW, g), measured after immersion of leaf petioles in demineralized water for 48 h in the dark at 4 °C; and dry weight (DW, g), measured after drying at 70 °C to a constant weight. Further, was calculated the relative water content, RWC (%) = (FW − DW)/(TW − DW) × 100.

### 2.3. Leaf Gas Exchange

Leaf gas exchange measurements were performed using a portable IRGA (LCpro+, ADC, Hoddesdon, UK), operating in the open mode. Measurements were performed on cloudless days under natural irradiance in two periods, morning (10:00 local time) and midday (13:30 local time). Net photosynthetic rate (A_n_, μmol CO_2_ m^−2^ s^−1^) and stomatal conductance (g_s_, mmol H_2_O m^−2^ s^−1^) were estimated using the equations developed by von Caemmerer and Farquhar [26]. Intrinsic water use efficiency was calculated as the ratio of A_n_/g_s_ (µmol mol^−1^).

### 2.4. Foliar Metabolic Assays

Total soluble sugars (SS) were extracted according to Irigoyen et al. [27], by heating the samples in ethanol/water (80/20, *v/v*) during 1 h, at 80 °C. Then, the soluble fractions were separated from the solid fraction. Starch (St) was extracted by heating the same solid fraction in 30% perchloric acid during 1 h, at 60 °C, according to Osaki et al. [28]. SS and St were analysed by reacting the obtained extract with fresh anthrone and placed in a boiling water bath for 10 min. Both SS and St concentration were expressed as mg g^−1^ DW, using glucose as a standard. Total soluble proteins (TSP) were quantified using the method of Bradford [29], by homogenizing the samples with a grinding medium (50 mM phosphate buffer (pH 7.8), 0.1 mM ethylenediaminetetraacetic acid (EDTA), 100 µM phenylmethylsulfonyl fluoride (PMSF) and 2% polyvinylpyrrolidone (PVP) (*w/v*). TSP were analysed by reacting the obtained extract with Bradford reagent and were expressed as mg g^−1^ DW using bovine serum albumin as a standard. Then, total thiols (–SH) in TSP extract were assessed according to Ellman [30], using 5,5′-dithiobis (2-nitrobenzoic acid) (DTNB). The concentration was calculated using an extinction coefficient of 13,600 M^−1^ cm^−1^, and was expressed as nmol mg^−1^ DW. Chlorophylls and carotenoids were extracted with acetone/water (80/20, *v/v*). Chlorophyll a (Chl_a_), chlorophyll b (Chl_b_), total chlorophyll (Chl_(a+b)_) and Chl_a_/Chl_b_ ratio were determined by using the formulas developed by Arnon [31] and Sesták et al. [32] and total carotenoids (Car) to the formulas developed by Lichtenthaler [33], and expressed as mg g^−1^ DW. Total reactive oxygen species (ROS) were determined with 2′,7′-dichlorofluorescein diacetate (DCFH-DA) (Sigma–Aldrich, Germany) [34]. A 25 mM solution was prepared in dimethyl sulphoxide for pending use. Twenty microliters of each sample were loaded into a small well ELISA plate containing 0.2 mL of PBS buffer (pH 7.4) and 12 µM of DCFH-DA and incubated for 20 min at 25°◦C. Fluorescence was measured at 485 nm and 530 nm (excitation and emission wavelength, respectively), in a CARY 50 Bio (Eclipse, Australia) every 15 min until 60 min after the incubation. 2′,7′,-dichlorofluorescein was used to obtained a calibration curve. Results were expressed as nmol DCF g^−1^ DW. H_2_O_2_ concentration were determined using a method described by Junglee et al. [35], with some modifications. The absorbance was measured at 350 nm and H_2_O_2_ was used to obtain a calibration curve. Results were expressed in µmol g^−1^ DW.

### 2.5. Biomass Accumulation Whole-Plant Water Use Efficiency and Mineral Analysis

Plants were harvested and total leaf area (WinDias image analysis system (Delta-T Devices Ltd., Cambridge, UK) and the dry weight of aboveground and belowground organs, after drying in a force-draft oven at 70 °C to a constant weight, were determined. Based on these data were determined the total biomass increase (TBI, %), the relative aboveground (RABI, %) and belowground (RBBI, %) biomass increase and total leaf area (TLA, cm^2^ plant^−1^). Leaf mass area (LMA, g m^−2^), net assimilation rate (NAR, g m^−2^ day^−1^, rate of biomass gain per leaf area) and leaf area per total plant biomass (LAR, m^2^ kg^−1^), were calculated using the equations proposed by Hunt [36], and the relative alleviation (RAI) and tolerance (RTI) indices estimated according to Gupta et al. [37]. Water use efficiency of biomass production (WUE_WP_, g kg^−1^) was determined, for each plant, by dividing total dry matter production by the cumulative amount of water used throughout the growing season. Total dry matter included the oven-dried leaves, stems and roots.

Following ground of dried plant samples (leaves, stems and roots), N concentration was determined by Dumas method in an elemental analyser (Primac, Skalar, The Netherlands). The concentrations of other elements (P, K, Ca, Mg, S, B, Fe, Cu, Zn, and Mn) were determined by ICP-OES (Quantima, GBC, Australia), after dry digestion and ash dissolution with HNO3 [38].

### 2.6. Statistical Analysis

All statistical calculations were performed using the software program SPSS for Windows (v. 22). After testing for ANOVA assumptions (homogeneity of variances with the Levene’s mean test, and normality with the Kolmogorov-Smirnov test), statistical differences were evaluated by one-way analysis of variance (ANOVA), followed by the post hoc Tukey’s test (*p* < 0.05). For statistical analysis of RWC, TBI, RABI and RBBI arcsine transformation was performed in percentage data.

## 3. Results

### 3.1. Leaf Gas Exchange and Water Status

**Figure 2:**
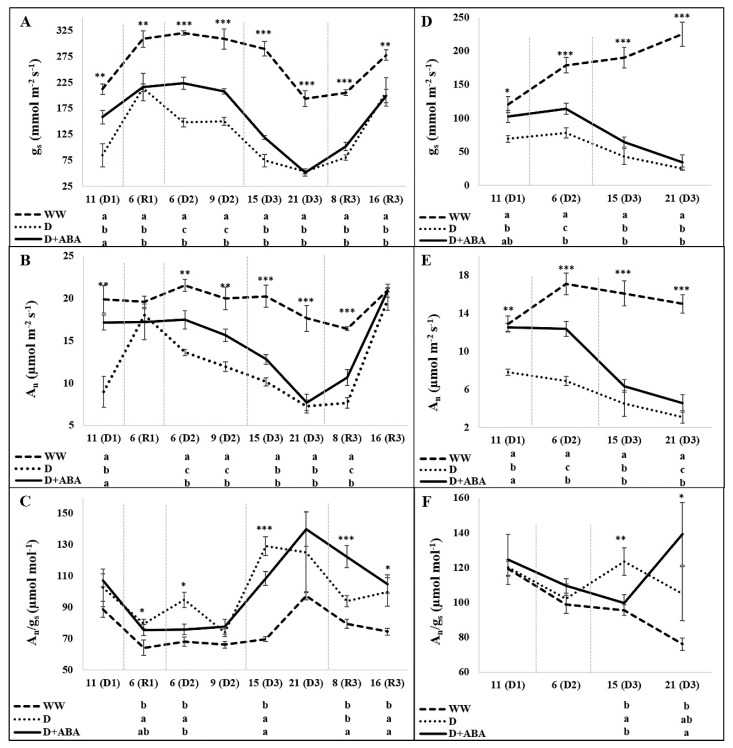
Evolution of stomatal conductance (g_s_), net photosynthetic rate (A_n_) and intrinsic water use efficiency (A_n_/g_s_) at morning (**A**–**C**) and midday (**D**–**F**) periods of days of drought (D1, D2, D3) and recovery (R1, R3) in each cycle, in leaves of well-watered (WW) and droughted without (D) and with abscisic acid (D + ABA) plants. Each point is average and vertical bars represent the S.E. (*n* = 8). Different letters indicate significant differences among treatments within each date (* *p* < 0.05, ** *p* < 0.01, *** *p* < 0.001).

In general, D and D + ABA plants presented lower A_n_ and g_s_ values than WW plants, while showed higher A_n_/g_s_ ratios (Figure 2).

A close performance of g_s_ and A_n_ was recorded, although small differences were identified. In opposite to D plants, eleven days after starting the 1st DP, g_s_ and A_n_ of D + ABA plants were statistically equal to WW plants, both during the morning and midday periods. After 6 days of rewatering was observed an increase of g_s_ in all treatments, but none of the droughted treatments reached the g_s_ of WW plants. By other side, during the 1st recovery period (RP) all plants exhibited similar A_n_. At the 6th and 9th days of the 2nd DP, D + ABA treatment exhibited higher g_s_ and A_n_ values than D plants, both at morning and midday. Nevertheless, the positive influence of ABA was lost 15 and 21 days after starting the 3rd DP, although an exception was observed at 21 days of 3rd DP during the midday period, where D + ABA showed higher A_n_ than D plants, in spite of the same g_s_. During the 16th days of the 3rd RP none of the droughted plants were able to full recovery g_s_, whereas a partial recovery of A_n_ was observed 8 days after rewatering, where D + ABA showed higher A_n_ than D plants, and a full recovery of A_n_ by droughted plants was recorded 16 days after rewatering. As a corollary of these trends, no significant differences on A_n_/g_s_ were recorded among treatments during the 1st DP, while D plants showed higher A_n_/g_s_ than WW plants 6 days after starting the 1st RP and superior efficiency than D + ABA and WW plants during the morning of 6(D2). Fifteen days after starting the 3rd DP, D and D + ABA plants showed higher A_n_/g_s_ than WW plants during the morning, while at midday only D plants kept this tendency. Later on, at midday of 21(D3), D + ABA plants presented higher A_n_/g_s_ than WW plants. During the last recovery period, D + ABA plants exhibited the highest A_n_/g_s_ at 8^th^ day, while at 16th day both D and D + ABA plants presented higher values than the well-watered treatment.

**Figure 3:**
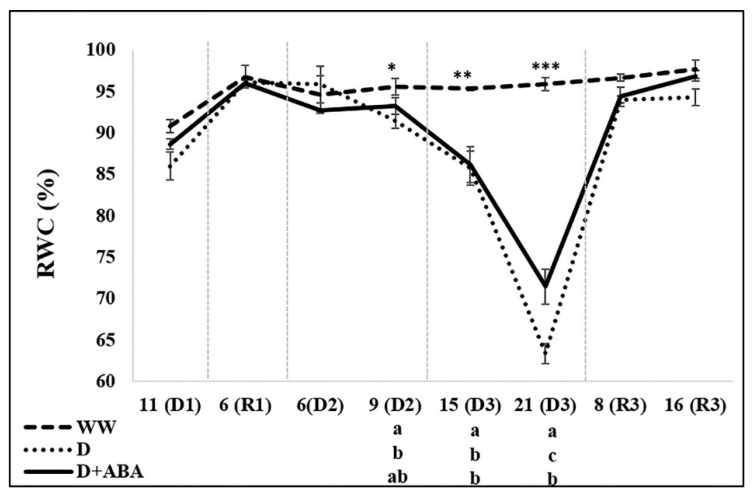
Changes of relative water content (RWC), of days of drought (D1, D2, D3) and recovery (R1, R3) in each cycle, in leaves of well-watered (WW) and droughted without (D) and with abscisic acid (D + ABA) plants. Each point is average and vertical bars represent the S.E. (*n* = 8). Different letters indicate significant differences among treatments within each date (* *p* < 0.05, ** *p* < 0.01, *** *p* < 0.001).

Plant water status was only influenced by the applied treatments on the 2nd and 3rd DPs (Figure 3).

Nine days after starting the 2nd DP, in opposite to the lower RWC values of D plants, D + ABA plants were able to keep similar RWC as WW plants. Moreover, fifteen days after starting the 3rd DP both D and D + ABA treatments showed lower RWC than WW plants, while after 21 days D + ABA plants presented a lower decrease of RWC than D plants.

### 3.2. Foliar Metabolic Responses

The results presented in Table 1 showed that both water regime and ABA modified the leaf metabolism, being the responses also reliant on the drought and rewatering events.

The drought imposition reduced the concentrations of TSS, TSP, -SH, Chl_(a+b)_ and Car in untreated plants, while ABA application countered this effect. By other side, while D treatment showed the higher St concentration, D + ABA plants had the lower accumulation. Furthermore, the Chl_a_/Chl_b_ ratio was lower in both D and D + ABA than in WW plants, whereas the Chl_(a+b)_/Car ratio was not significantly different among treatments. Total ROS and H_2_O_2_ accumulation were differently affected by the applied treatments, as drought increased total ROS accumulation by 50% in D and only 25% in D + ABA plants, while it reduced H_2_O_2_ accumulation in D plants.

After 8 days of rewatering, D plants still exhibited lower TSS accumulation than D + ABA plants, and D + ABA plants still displayed the lower St accumulation in their leaves. Relatively to the drought period, the concentration of TSP and -SH decreased during the recovery phase in D + ABA plants, explaining the similar and lower values relatively to D and WW plants, respectively. Meanwhile, D plants still presented higher accumulation of ROS, whereas enhanced H_2_O_2_ concentration to WW levels, which was lower than in D + ABA plants. Regarding the concentrations of photosynthetic pigments, the influence of treatments felt during the drought period was reversed by rewatering.

### 3.3. Growth Biomass Accumulation and Water Use Efficiency and Mineral Dynamics

The increase of plant biomass trough the experimental period was reduced in D plants (Table 2), namely due to lower RABI, while the application of ABA countered the drought effect, mainly due to a tendency to higher RBBI, being the variation of TBI strictly associated with the responses of NAR. TLA was reduced by drought in both D and D + ABA plants, while were observed higher LMA and lower LAR in D + ABA treatment. As a corollary of the previous changes, was clear that the application of ABA strongly ameliorated both RTI and RAI to WW levels and improved WUE_WP_ 4.2 and 2 times in relation to WW and D treatments, respectively (Table 2).

With the exception of nitrogen, calcium, iron and zinc, and dependent on the plant organ, the concentrations of other minerals were somehow affected by water availability and ABA application (Table 3). Phosphorous was reduced by ABA in leaves of droughted plants and by drought in stems of D treatment. Potassium was reduced in leaves by drought and, specially, in combination with ABA, while the drought-induced reduction of K in roots was annulled by ABA application. Magnesium and manganese concentrations increased, and sulphur decreased in stems of D plants. Boron increased in roots of D + ABA plants, while drought reduced the Cu concentration in roots of both D and D + ABA plants. On the other hand, water availability and ABA application affected the minerals allocation patterns, with the exception of calcium, magnesium, zinc and manganese (Table 4).

Drought reduced the allocation of nitrogen into leaves and of sulphur into stems of both D and D + ABA plants, while enhanced the allocation of N into roots and of phosphorous into leaves and roots, at the expenses of stems, of D plants. The application of ABA improved the allocation of potassium into roots in relation to WW and D treatments. Boron allocation into roots increased, at the expenses of leaves, in both D and D + ABA plants. Drought reduced the allocation of iron into leaves of D plants and the allocation of copper into roots of D + ABA treatment.

## 4. Discussions

### 4.1. ABA Pre-Treatment Improved the Physiological and Metabolic Functions of Olive Trees During Successive Drought Events

Stomatal closure is one of the first lines of defence against immediate dehydration, although at the expense of CO_2_ supply to photosynthesis [39]. The foliar spray of ABA before withholding water delayed the drought effects on g_s_ at the mid-term (Figure 2A). As ABA-induced stomatal closure is one of the best known and assumed responses of this hormone [13,15,16,20], our results were somehow unexpected. Exogenous ABA is rapidly metabolized to biologically inactive products [40], so we hypothesized that a transient effect of ABA might occurred, with a g_s_ reduction during the first days after the application which were no longer visible 11 days after starting the 1st DP. Moreover, this likely induced conservative behaviour allowed the sprayed plants to save water relatively to the control and, therefore, to maintain the cellular machinery to work properly. This hypothesis is supported by the results of Du et al. [23], who described a significant decrease of g_s_ on the first day after ABA application in plants subjected to drought, followed by a slow decrease that was reversed after 6 days. In the same way, He et al. [20], described that ABA application induced a transient increase in g_s_ after a reduction during the initial phase of drought. Agehara and Leskovar [15] also described a stomatal reopening resulting from degradation of exogenous ABA under well-watered conditions, and a delay in stomatal closure after an instantaneous decline upon ABA application under drought conditions.

Despite D + ABA plants were able to keep higher g_s_ than D plants until the end of the 2nd DP (Figure 2A), they exhibited similar or even higher values of RWC (Figure 3). In fact, ABA was effective to slow down the RWC decline under severe drought, with a decline of 25% against the decrease of 34% of D plants 21 days after starting the 3rd DP (Figure 3). Relative water content is probably the most appropriate measure of plant water status in terms of the physiological consequence of cellular water deficit, as it integrates leaf water potential with the effect of a powerful mechanism of conserving cellular hydration, the osmotic adjustment [41]. The accumulation of compatible solutes in cells is a common response of plants under drought, which lowered the osmotic potential and attracts water molecules into the cells and ultimately maintains cells turgor [2]. In the present study, the higher concentration of soluble sugars, important compatible solutes, found in D + ABA relatively to D plants (Table 1), might contributed to superior osmotic adjustment capacity of ABA-sprayed plants, and are also involved in detoxification of ROS and stabilization of cellular macromolecules structures [42]. On the other hand, starch, being temporally stored, acts mostly as a reservoir of carbon for future use, depending on the source-sink dynamics concept. The higher starch contents in D leaves (Table 1), in spite of lower A_n_, suggest that carbon was not translocated out of the leaves, reflecting an excess supply relative to demand. Conversely, the lower starch concentrations in in D + ABA leaves may be linked to tissue osmotic adjustment, as one of the main sources of osmolytes are the starch reserves, which supply soluble sugars. Thus, the application of ABA contributed to minimize drought stress by maintaining turgid leaves, an important response to improve drought tolerance, as plant metabolic processes are more dependent on turgor than to absolute water potential [41]. Previous studies also described the role of exogenous ABA on osmotic adjustment induction and/or carbohydrates accumulation [17,20,43,44] and on RWC improvement [13,15,20,23].

One of the most important metabolic processes affected by drought is photosynthesis [1,10,39]. The registered positive effect of ABA on photosynthetic responses was mainly due to the lower stomatal limitations, although a reduction of non-stomatal limitations was also evident on 21(3D), at midday, with higher A_n_ accompanied by the same g_s_ in D + ABA plants (Figure 2A,B). The influence of exogenous ABA on reducing the drought effects on photosynthesis was also described in other studies, either due to reduced stomatal or/and non-stomatal limitations [16,17,20,22]. The higher A_n_ recorded on 21(3D), at midday, could also be related to the higher concentrations of photosynthetic pigments (Table 1), as in other studies [15,18,22], because the maintenance of better water status by the application of ABA might minimize chlorophylls loss by dehydration. Furthermore, the concentration of carotenoids was higher on D + ABA than on D plants, due to a lower oxidative stress, and as carotenoids are precursors of ABA [45], probably D + ABA plants might not need to use those pigments to produce ABA at the same extent as D plants, attesting by the reduced stress level that those plants experience. Carotenoids besides to act as a light-harvesting pigments, are involved in excess energy dissipation and in ROS scavenging [46], also protecting the highly susceptible chlorophylls from oxidative damage.

Interestingly, despite the low RWC values (Figure 3), D + ABA plants were able to keep the concentration of TSP at WW levels (Table 1), which may have been achieved by reduced degradation and/or by the induction of stress response proteins by ABA signalling. In fact, beyond accumulation of compatible solutes, which are known to scavenge reactive species and protect cellular membranes and proteins [46], D + ABA plants exibithed better water status (Figure 3) and lower ROS accumulation than D plants (Table 1). The higher concentration of -SH is also a signal of lower oxidative damage, as thiol groups are one of the more susceptible targets in proteins, which when suffer from irreversible oxidation can seriously damage proteins [47]. On the other side, it was reported that ABA application enhances the expression and/or the activity of resistance proteins and antioxidant enzymes [12,13,14,18]. Moreover, the negative association between total ROS and H_2_O_2_ concentration in D + ABA plants indicates an efficient detoxification of highly reactive species, as H_2_O_2_ is the less reactive and the product of another ROS detoxification [48]. Likewise, Li et al. [14] also described higher H_2_O_2_ accumulation in droughted plants sprayed with ABA, although only up to certain duration and severity of the stress. Furthermore, H_2_O_2_ is also a potent signaling molecule, due to its long half-life and the ability to cross cellular membranes, which leads to the activation of antioxidant enzymes [49].

### 4.2. ABA Pre-Treatment Allowed a Fast Recovery of Net Photosynthesis upon Rewatering

Information regarding the influence of ABA pre-treatment in drought recovery plant responses is scarce. Even if direct effects of ABA do not persist too longer, the influence of hormone could be felt as the plants’ capacity for recovery from previous drought is dependent on the severity of the damages caused by the previous stress. Upon rewatering, olive tree is known to have a conservative behaviour in terms of g_s_ reestablishment [8,50,51], improving water conservation for ensuing drought events. In fact, none of the stressed treatments completely restored the g_s_ in both RPs (Figure 2A). Nonetheless, following the typical olive tree response, water status recovered earlier than g_s_, as in other studies [9,52]. Similarly, ABA pre-treatment did not affect the RWC reestablishment (Figure 3), suggesting that the difference in the extent of damages registered during the drought events was not sufficient to disturb the capacity to uptake the newly available water. Olive tree was described to recover its water status faster than other fruit trees, even if it shows a slow recovery of stomatal conductance [53]. On the other hand, despite the limitation in g_s_ restoration, A_n_ full recovered upon rewatering, although depending on stress severity and physiological status (Figure 2B). Following a mild drought event, observed during the 1st DP, both D and D + ABA plants showed an early recovery of A_n_ than following the severest drought event, registered during the 3rd DP. In addition, the protection induced during the severe drought phase allowed the fast recovery of A_n_ on D + ABA relatively to D plants. Meanwhile, the concentration of photosynthetic pigments was restored in D plants (Table 1), while remained relatively constant in D + ABA plants after rewatering, confirming that those molecules were better protected against drought in ABA-sprayed plants. Conversely, the reduction on the concentration of TSP in D + ABA plants after rewatering (Table 1) reinforces the thesis of ABA-responsive proteins, suggesting that ABA influence persists, at least, in gene expression. Although 8 days after the beginning of the 3rd recovery period D + ABA plants had not yet displayed a full recovery of A_n_ (Figure 2B), they maintained higher concentration of TSS, probably at the expense of starch reserves, which decreased with rewatering (Table 1). This response might accomplish the demand for a rapid recovery of physiological functions and growth [54] and suggests that ABA pre-treatment allowed to divert a higher proportion of the newly assimilated carbon into soluble sugar export for plant growth, and less to temporary storage, as starch [55]. Thus, these results with the addition of ABA support the assumption that growth of trees under drought and rewatering cycles are positively associated with higher soluble sugars/starch ratio, and not to higher nonstructural carbohydrates concentration. Meanwhile, after stress relief, D + ABA meet the ROS levels of WW plants, continuing lower than on D plants, while showed the highest concentration of H_2_O_2_, suggesting an efficient detoxification of ROS by these plants, especially the highly reactive.

### 4.3. ABA Pre-Treatment Modulates Olive Tree Ionome after Successive Drought-Rewatering Cycles

Limited nutrient uptake and reduced minerals concentrations is a regular response to lower water availability [56]. However, in our study a minor reduction in minerals concentrations were recorded (Table 3) in D plants, probably due to a concentration effect derived from the lower production of biomass, while in D + ABA plants the response was also minimized due to the tendency for higher investment on root biomass (Table 2) and on higher stomatal conductance, which maintained the transpiration stream and, thus, the absorption of minerals. Changes in the allocation patterns among organs were also responsible for shifts in minerals concentrations in water-stressed plants, with special impact on N, P, K, S and B. The nutrient allocation reflects the balance between the capacity to obtain, transport and store nutrients [57], being also dependent on where and how nutrients are used by the plant, and whether this pattern is changed under atypical conditions [58]. In this way, the different allocation patterns suggest a selective behavior according to the plant needs, as presented below. All drought-stressed plants maintained the concentrations of N similar to WW levels (Table 3) by improving the N allocation to roots in detriment of leaves (Table 4), probably to optimize the shoot/root ratio, in a strictly association to the important role of N in cell division and, thus, root growth promoting [59]. Furthermore, adequate nitrogen concentrations reduce oxidative stress and membrane damage under drought stress through physio-biochemical adjustments, including higher level of nitrogenous compounds, up-regulation of N-associated metabolic enzymes activities, and higher accumulation of osmolytes [60]. Meanwhile, the lower S concentration on stems of droughted plants, associated with a lower allocation of S to stems (Table 3 and Table 4), suggest that S was preferentially allocated to leaves and roots, in order to meet metabolic functions as ROS detoxification, since the tripeptide glutathione is an important sulphur compound [61]. In addition, sulphur is of great significance as structural component of proteins and functioning of enzymes, as well vitamins and cofactors (biotin, thiamine, CoA, and *S*-adenosyl-Met), and a variety of secondary products [62]. Interestingly, D plants presented higher allocation of P to leaves and roots, at the expenses of stems (Table 4), to adjust metabolic processes, as P is a major component of nucleic acids, membrane lipids, and phosphorylated intermediates of energy metabolism, being also involved in controlling key enzyme reactions and on photosynthesis [63,64]. Conversely, ABA-sprayed plants, in spite of inferior leaf P concentration, although in adequate range, had allocation patterns similar to well-watered plants, with major allocation stems, as P allocated to stems play important roles in respiration, internal nutrient recycling, and photosynthate loading and export in the phloem to meet the higher demand for photosynthate transport [65]. On the other hand, in spite of higher B concentrations in roots of all droughted plants, only in D + ABA treatment this difference was statistically significant in relation to WW plants (Table 3). Nonetheless, both D and D + ABA plants enhanced the B allocation to roots in detriment of leaves (Table 4), claiming the major functions of B on the structure on cell walls and on regulation of carbohydrates metabolism, promoting root cell elongation and, thus, root growth [66]. Furthermore, the application of ABA potentiated the drought-induced reduction of K concentration in leaves. However, by other side, ABA was effective to maintain an adequate concentration of K in roots (Table 3), in association with higher allocation of K to roots (Table 4), which has a significant relevance, as K is a principal cation in vacuoles, contributing to osmotic adjustment and, thus, to increased expansion of cells via high cell turgor pressure [61,67]. Thus, these results support the assumption of Sardans and Peñuelas [68] that the patterns of allocation of nutrients allow an optimum trade-off between the rate of growth and the capacity of tolerance to stress [68].

### 4.4. ABA Pre-Treatment Attenuated the Growth Inhibition and Improved the Water Use Efficiency after Successive Drought-Rewatering Cycles

The reduction in leaf area under water deficit (Table 2), is a common response of plants caused by a decrease in cell turgor, which reduces the driving force for cell expansion [69]. On the other hand, in spite of higher RWC in ABA-treated than in D plants during the peak stress (Figure 2), no significant differences were record in TLA (Table 2), suggesting that, under the present environmental conditions and with three rewatering cycles, D plants did not need more drastic measures to control plant water losses, such as reduction in leaf area. In addition, our results indicate that the stress alleviation by ABA was not mediated by leaf area adjustment, as in the study of Agehara and Leskovar [15]. Nevertheless, ABA-treated plants presented higher investment of photosynthates in assimilation area, as evidenced by the higher LMA, at whole-plant level, relatively to D plants (Table 2), as a result of changes in leaf chemical composition (at least, higher concentrations of total nonstructural carbohydrates and total soluble proteins) and the cumulative effects, even if not significant, of superior mean leaf thickness (493.4 μm against 488.1 μm in mature leaves) and a lower proportion of leaves that are still in the expansion phase (10.8% against 12.5%). Despite the existence of few studies on the effect of hormones, previous works indicated the increase of LMA with the application of cytokinins [70], while the addition of gibberellins decreased LMA [71]. Meanwhile, tomato mutants deficient in ABA presented high LMA that was associated with their deteriorated water status [72]. Higher values of LMA of ABA-treated plants has profound physiological and ecological consequences, including nutrient retention, protection from desiccation and greater lifespan of leaves and roots [73,74,75].

The reduction in leaf area did not limit significantly the carbon acquisition by D + ABA plants, as happened in D treatment. In fact, the ABA-induced protection of cellular machinery and the improved physiological and biochemical functions, contributed to decrease the drought-induced growth inhibition, as demonstrated by the 26.8%, 27.1% and 39.2% higher TBI, RABI and RBBI, respectively (Table 2). The alleviation of drought-inhibition in growth and biomass accumulation was also documented in other studies [19,24,44], although a negative and/or the absence of influence were also observed [15,23]. The alleviation of drought effects by ABA pre-treatment, as demonstrated by similar TBI values of WW plants, was associated with a different biomass partitioning among plant organs, as D + ABA plants increased the dry matter allocation to belowground in detriment of aboveground organs (Table 2). This response is another important mechanism to tolerate repeated cycles of drought [76], although to be transversal to both D and D + ABA plants were more pronounced with ABA application. By optimizing shoot/root ratio, these plants maintained the equilibrium between water supply and demand and improved the partitioning of assimilates to recovery, after mobilization of stored reserves in roots [9]. Moreover, the stimulation of root development by ABA addition was previously described under drought conditions [19,44].

The carbon balance of plants during periods of drought and recovery depends on the velocity and degree of photosynthetic recovery, as well on the degree and velocity of photosynthesis decline during water depletion [77]. The higher biomass accumulation in D + ABA plants relatively to D plants was associated with the greater NAR (Table 2), which is related to the enhanced A_n_ observed, especially during the drought events and also in the first days of the recovery event (Figure 2A,C). Furthermore, as previously discussed, the higher TSS concentrations in D + ABA than in D plants during both DP and RP, might contributed to osmotic adjustment, maintaining turgor and meristem viability, and serve as the primary source of carbohydrates, enhancing rapid regrowth of plants after rewatering [3,54]. As total leaf area of droughted plants was not affected by ABA, while TBI increased, LAR was lowered by the ABA application (Table 2). In addition, plant biomass production depends on the amount of water used for growth, as well on water use efficiency [78]. Remarkably, ABA improved WUE_WP_ by 320% and by 108% in relation do WW and D conditions, respectively (Table 2). Meanwhile, the absence of a significant association between A_n_/g_s_ and WUE_WP_, namely amongst D and D + ABA treatments, reflects the spatial and temporal variations, since A_n_/g_s_ denotes responses of individual leaves at specific environmental conditions, while WUE_WP_ represents the whole-plant carbon and biomass acquisition per unit of water used along all the growth season, integrating other physiological processes like respiration and night transpiration processes [79]. As in this study, He et al. [20] reported that ABA increased water use efficiency for grain yield in soybean under drought conditions. The modulation of this trait is of extreme importance for production on relatively dry sites, as plants that display high WUE_WP_ appear to be the most promising [7]. As a corollary, the RTI and RAI calculated values indicate that ABA was effective to help olive to offset drought.

## 5. Conclusions

**Figure 4:**
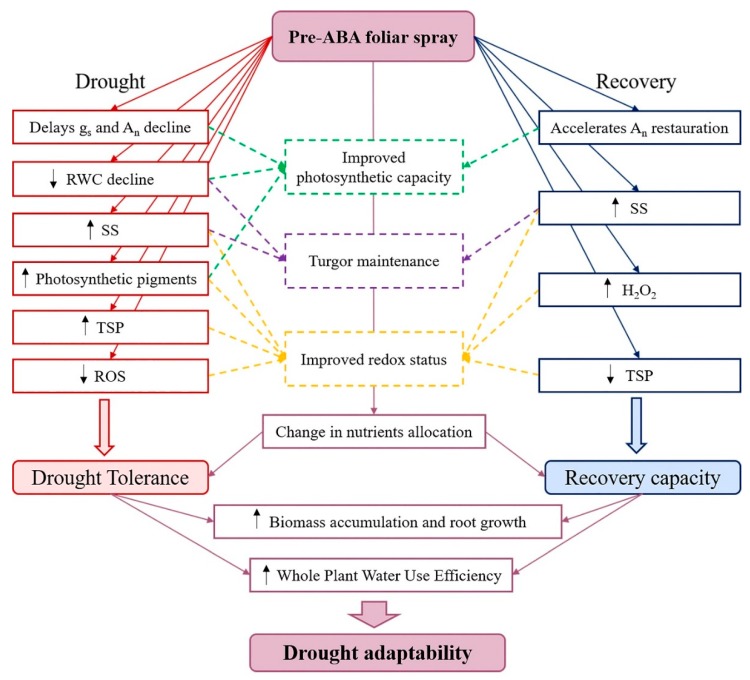
Abscisic acid-mediated responses of olive trees under drought and recovery events. Abbreviations: g_s_, stomatal conductance; A_n_, net photosynthetic rate; RWC, relative water content; SS, soluble sugars, TSP, total soluble proteins; ROS, reactive oxygen species; H_2_O_2_, hydrogen peroxide.

The foliar application of ABA before withholding water improved both drought tolerance and recovery capacity, i.e., drought adaptability, of young olive trees in outdoor conditions, as illustrated in Figure 4. The addition of ABA contributed to delay the negative drought effects on g_s_ and A_n_, to improve the turgor maintenance associated with osmotic adjustment and to reduce the drought-induced water status decline and oxidative stress. Even if not directly, ABA application improved the physiological and biochemical functions during the recovery, as the plant’s capacity of recovery from previous drought is dependent on the severity of the damages caused by the previous stress. The modulation of ionome in ABA-sprayed plants also suggests that the hormone changed the nutrients requirement and their allocation among the different plant organs, with special influence in K, N and B accumulation in roots. As a consequence, the ABA-triggered changes in plant functions attenuated the drought-induced decline in biomass accumulation, induced root growth and enhanced water use efficiency after successive drought-rewatering cycles. These changes are likely to be of real adaptive significance, with important implications for plant growth and productivity when plants are exposed to challenging environments, as commonly occurs in Mediterranean ecosystems. Moreover, as the experiment allowed the plants to growth under near-natural circumstances, the results might be transferable to field conditions.

## Figures and Tables

**Table 1:** Oxidative stress indicators and foliar metabolites concentrations of well-watered (WW) and droughted without (D) and with abscisic acid (D + ABA) plants during the 3rd drought (D3) and recovery (R3) periods. Total soluble sugars (TSS, mg g^−1^ DW), starch (St, mg g^−1^ DW), total soluble proteins (TSP, mg g^−1^ DW), total thiols (-SH, nmol mg^−1^_DW_), total chlorophylls (Chl_(a+b),_ mg g^−1^ DW) and total carotenoids (Car, mg g^−1^ DW), and the ratio of chlorophyll a/chlorophyll b (Chl_a_/Chl_b_) and chlorophylls/carotenoids (Chl_(a+b)_/Car), total reactive oxygen species (ROS, nmol g^−1^ DW) and H_2_O_2_ (µmol g^−1^ DW).

		WW	D	D + ABA	Sig.
TSS	**21 (D3)**	235.2 ± 6.6 ^a^	203.1 ± 5.9 ^b^	225.3 ± 5.2 ^a^	**
	**8 (R3)**	258.6 ± 5.6 ^ab^	217.1 ± 13.5 ^b^	264.0 ± 5.1 ^a^	**
St	**21 (D3)**	49.4 ± 5.8 ^ab^	63.7 ± 5.7 ^a^	41.1 ± 3.2 ^b^	*
	**8 (R3)**	54.2 ± 6.3 ^a^	52.8 ± 6.4 ^a^	32.2 ± 1.5 ^b^	*
TSP	**21 (D3)**	6.69 ± 0.34 ^a^	2.59 ± 0.12 ^b^	6.77 ± 0.31 ^a^	***
	**8 (R3)**	7.95 ± 0.49 ^a^	3.75 ± 0.13 ^b^	4.22 ± 0.88 ^b^	***
-SH	**21 (D3)**	1.88 ± 0.07 ^a^	1.03 ± 0.07 ^b^	1.66 ± 0.02 ^a^	***
	**8 (R3)**	2.95 ± 0.46 ^a^	1.14 ± 0.04 ^b^	1.40 ± 0.08 ^b^	***
Chl_(a+b)_	**21 (D3)**	3.04 ± 0.09 ^a^	2.58 ± 0.11 ^b^	2.92 ± 0.05 ^a^	**
	**8 (R3)**	3.25 ± 0.05	3.31 ± 0.06	3.11 ± 0.10	n.s.
Car	**21 (D3)**	0.678 ± 0.015 ^a^	0.600 ± 0.03 ^b^	0.665 ± 0.01 ^a^	**
	**8 (R3)**	0.706 ± 0.033	0.700 ± 0.022	0.697 ± 0.005	n.s.
Chl_a_/Chl_b_	**21 (D3)**	3.14 ± 0.09 ^a^	2.88 ± 0.16 ^b^	2.91 ± 0.02 ^b^	**
	**8 (R3)**	3.13 ± 0.07	2.91 ± 0.15	2.97 ± 0.03	n.s.
Chl_(a+b)_/Car	**21 (D3)**	4.48 ± 0.05	4.30 ± 0.05	4.40 ± 0.05	n.s.
	**8 (R3)**	4.60 ± 0.15	4.73 ± 0.23	4.46 ± 0.18	n.s.
ROS	**21 (D3)**	0.289 ± 0.021 ^c^	0.435 ± 0.020 ^a^	0.363 ± 0.025 ^b^	***
	**8 (R3)**	0.371 ± 0.025 ^b^	0.543 ± 0.037 ^a^	0.373 ± 0.006 ^b^	**
H_2_O_2_	**21 (D3)**	25.0 ± 0.1 ^a^	13.7 ± 0.2 ^b^	26.0 ± 0.1 ^a^	***
	**8 (R3)**	18.9 ± 0.5 ^b^	17.4 ± 0.5 ^b^	25.6 ± 0.1 ^a^	***

Values are means ±SE. Different letters indicate significant differences among treatments within each date (ns, not significant, * *p* < 0.05, ** *p* < 0.01, *** *p*< 0.001).

**Table 2:** Total biomass increase (TBI, %), relative aboveground biomass increase (RABI, %), relative belowground biomass increase (RBBI, %), total leaf area (TLA, cm^2^ plant^−1^), net assimilation rate (NAR, g m^−2^ day^−1^), leaf area per total plant biomass (LAR, m^2^ kg^−1^), leaf mass area (LMA, g m^−2^), relative tolerance index (RTI, %), relative alleviation index (RAI, %) and the whole-plant water use efficiency (WUE_WP_, g kg^−^^1^) of well-watered (WW) and droughted without (D) and with abscisic acid (D + ABA) plants.

	WW	D	D + ABA	Sig.
TBI	64.2 ± 6.2 ^a^	25.4 ± 5.6 ^b^	53.0 ± 6.6 ^a^	**
RABI	88.8 ± 7.1 ^a^	35.6 ± 6.7 ^c^	62.7 ± 9.3 ^b^	**
RBBI	79.7 ± 7.2	59.3 ± 6.0	98.5 ± 8.2	+
TLA	1398.9 ± 59.2 ^a^	985.7 ± 53.1 ^b^	1090.0 ± 89.2 ^b^	**
NAR	6.11 ± 0.41 ^a^	2.89 ± 0.59 ^b^	5.78 ± 0.55 ^a^	**
LAR	1.15 ± 0.02 ^a^	1.09 ± 0.02 ^a^	1.05 ± 0.02 ^b^	**
LMA	234.0 ± 3.8 ^b^	235.9 ± 4.9 ^b^	258.5 ± 2.7 ^a^	**
RTI	100.0 ± 2.3 ^a^	74.1 ± 2.8 ^b^	86.6 ± 4.0 ^a^	**
RAI	134.8 ± 3.7 ^a^	100.0 ± 3.4 ^b^	125.7 ± 5.2 ^a^	**
WUE_WP_	2.02 ± 0.08 ^b^	4.07 ± 0.59 ^b^	8.49 ± 1.02 ^a^	***

Values are means ±SE. Different letters within a line demonstrate significant differences between treatments (ns, not significant, + 0.1 < *p* > 0.05, ** *p* < 0.01, *** *p* < 0.001).

**Table 3:** Minerals content (g kg^−1^ DW for macronutrients, and mg kg^−1^ DW for micronutrients) in the different plant organs of well-watered (WW) and droughted without (D) and with abscisic acid (D + ABA) plants at the end of the experiment.

	Minerals Content			Sig.
	WW	D	D + ABA	
N_Leaf_	19.1 ± 0.7	18.8 ± 0.7	18.1 ± 0.9	ns
N_Stem_	5.16 ± 0.20	5.82 ± 0.28	5.77 ± 0.24	ns
N_Root_	14.8 ± 0.5	14.9 ± 0.8	14.2 ± 0.7	ns
P_Leaf_	3.94 ± 0.21 ^a^	3.89 ± 0.19 ^a^	3.01 ± 0.20^b^	*
P_Stem_	2.99 ± 0.18 ^a^	1.62 ± 0.29 ^b^	2.57 ± 0.33 ^ab^	*
P_Root_	2.08 ± 0.31	1.98 ± 0.38	1.85 ± 0.22	ns
K_Leaf_	13.5 ± 0.6 ^a^	11.2 ± 0.2 ^b^	8.33 ± 0.62 ^c^	***
K_Stem_	5.95 ± 1.5	5.56 ± 0.51	4.89 ± 1.33	ns
K_Root_	8.31 ± 0.41 ^a^	3.59 ± 0.56 ^b^	7.32 ± 0.54 ^a^	***
Ca_Leaf_	3.10 ± 0.31	3.50 ± 0.89	3.27 ± 0.79	ns
Ca_Stem_	1.78 ± 0.52	1.98 ± 0.51	1.01 ± 0.27	ns
Ca_Root_	2.21 ± 0.48	2.47 ± 0.89	2.59 ± 0.19	ns
Mg_Leaf_	0.416 ± 0.089	0.362 ± 0.098	0.450 ± 0.056	ns
Mg_Stem_	0.236 ± 0.068 ^b^	0.453 ± 0.071 ^a^	0.241 ± 0.042 ^b^	*
Mg_Root_	1.56 ± 0.18	1.45 ± 0.19	1.67 ± 0.11	ns
S_Leaf_	3.24 ± 0.18	3.62 ± 0.30	3.26 ± 0.27	ns
S_Stem_	0.307 ± 0.041 ^a^	0.129 ± 0.039 ^b^	0.186 ± 0.032 ^ab^	*
S_Root_	0.715 ± 0.294	0.751 ± 0.154	0.880 ± 0.131	ns
B_Leaf_	25.3 ± 1.5	23.0 ± 2.1	25.0 ± 1.5	ns
B_Stem_	24.1 ± 0.9	28.1 ± 2.2	28.2 ± 3.7	ns
B_Root_	30.1 ± 1.8 ^b^	38.8 ± 2.9 ^ab^	45.4 ± 4.3 ^a^	*
Fe_Leaf_	15.1 ± 2.1	10.4 ± 1.1	19.0 ± 4.0	ns
Fe_Stem_	10.8 ± 2.0	10.9 ± 2.5	7.3 ± 1.4	ns
Fe_Root_	295.3 ± 23.5	288.5 ± 39.8	363.0 ± 32.0	ns
Zn_Leaf_	17.7 ± 3.0	16.3 ± 2.2	14.8 ± 1.4	ns
Zn_Stem_	10.9 ± 1.2	13.2 ± 1.8	10.2 ± 3.1	ns
Zn_Root_	50.1 ± 4.8	42.0 ± 7.2	52.0 ± 8.4	ns
Mn_Leaf_	29.5 ± 6.2	23.5 ± 5.8	48.6 ± 12.1	ns
Mn_Stem_	5.01 ± 0.10 ^b^	8.82 ± 1.31 ^a^	5.42 ± 1.79 ^b^	*
Mn_Root_	48.2 ± 5.6	51.9 ± 12.2	50.1 ± 7.7	ns
Cu_Leaf_	12.5 ± 0.9	11.5 ± 1.8	15.8 ± 4.2	ns
Cu_Stem_	9.01 ± 1.21	9.97 ± 1.99	8.51 ± 1.57	ns
Cu_Root_	21.2 ± 1.8 ^a^	12.2 ± 1.9 ^b^	8.23 ± 2.28 ^b^	***

Values are means ± SE. Different letters indicate significant differences among treatments (n.s.—not significant, * *p* < 0.05, ** *p* < 0.01, *** *p* < 0.001).

**Table 4:** Minerals allocation (%) by the different plant organs of well-watered (WW) and droughted without (D) and with abscisic acid (D + ABA) plants at the end of the experiment.

	Minerals Allocation			Sig.
	WW	D	D + ABA	
N_Leaf_	49.3 ± 0.3 ^a^	39.8 ± 1.2^b^	40.1 ± 2.5^b^	*
N_Stem_	25.8 ± 0.9	25.3 ± 1.2	26.4 ± 0.8	ns
N_Root_	24.9 ± 0.9 ^b^	34.9 ± 2.6 ^a^	33.5 ± 2.4 ^ab^	*
P_Leaf_	30.4 ± 1.9 ^b^	39.2 ± 1.1 ^a^	26.2 ± 1.9 ^b^	**
P_Stem_	55.5 ± 0.6 ^a^	33.2 ± 4.7 ^b^	53.6 ± 2.9 ^a^	**
P_Root_	14.1 ± 2.0 ^b^	27.6 ± 4.8 ^a^	20.1 ± 2.0 ^ab^	*
K_Leaf_	39.6 ± 5.5	42.4 ± 2.1	32.6 ± 6.5	ns
K_Stem_	43.2 ± 6.8	40.7 ± 2.9	36.4 ± 8.3	ns
K_Root_	17.2 ± 1.0 ^b^	16.9 ± 3.8 ^b^	31.0 ± 2.9 ^a^	*
Ca_Leaf_	46.3 ± 6.3	36.8 ± 7.0	40.8 ± 3.7	ns
Ca_Stem_	28.9 ± 7.5	36.4 ± 5.1	22.7 ± 4.2	ns
Ca_Root_	24.8 ± 4.3	26.8 ± 81	36.5 ± 3.3	ns
Mg_Leaf_	19.8 ± 3.9	15.2 ± 1.3	17.2 ± 1.5	ns
Mg_Stem_	20.1 ± 5.0	27.0 ± 5.6	18.0 ± 2.5	ns
Mg_Root_	60.1 ± 3.6	57.8 ± 3.5	64.7 ± 3.1	ns
S_Leaf_	68.5 ± 2.0	73.5 ± 3.22	68.3 ± 1.8	ns
S_Stem_	15.1 ± 1.1 ^a^	8.20 ± 1.25 ^b^	9.3 ± 1.5 ^b^	**
S_Root_	16.4 ± 0.9	18.3 ± 3.3	22.4 ± 2.6	ns
B_Leaf_	30.2 ± 0.9 ^a^	20.6 ± 0.6 ^b^	19.9 ± 1.1 ^b^	***
B_Stem_	44.0 ± 0.9	42.5 ± 1.2	44.3 ± 2.6	ns
B_Root_	25.8 ± 0.4 ^b^	36.9 ± 0.8 ^a^	35.8 ± 2.3 ^a^	***
Fe_Leaf_	7.28 ± 0.9 ^a^	3.82 ± 0.61 ^b^	4.08 ± 0.71 ^ab^	*
Fe_Stem_	8.05 ± 2.9	9.11 ± 5.9	3.72 ± 0.63	ns
Fe_Root_	84.7 ± 3.0	87.1 ± 1.2	92.2 ± 0.9	ns
Zn_Leaf_	19.8 ± 1.6	18.4 ± 2.3	16.3 ± 2.1	ns
Zn_Stem_	23.8 ± 2.8	28.9 ± 7.1	22.1 ± 5.0	ns
Zn_Root_	56.4 ± 2.8	52.7 ± 6.2	61.6 ± 3.7	ns
Mn_Leaf_	40.3 ± 3.7	35.2 ± 5.3	37.0 ± 4.6	ns
Mn_Stem_	16.8 ± 3.9	18.9 ± 2.2	12.2 ± 2.8	ns
Mn_Root_	42.9 ± 8.1	54.1 ± 3.1	50.8 ± 4.3	ns
Cu_Leaf_	30.1 ± 3.2	35.2 ± 3.4	38.5 ± 6.2	ns
Cu_Stem_	34.1 ± 3.2	39.6 ± 3.5	40.4 ± 4.5	ns
Cu_Root_	35.8 ± 2.9 ^a^	25.2 ± 2.8 ^ab^	21.0 ± 4.7 ^b^	*

Values are means ± SE. Different letters indicate significant differences among treatments (n.s.—not significant, * *p* < 0.05, ** *p* < 0.01, *** *p* < 0.001).
